# Opening and Drainage of Cavities in the Lungs

**Published:** 1882-02

**Authors:** 


### OPENING AND DRAINAGE OF CAVITIES IN THE LUNGS.

---

Drs. Fenger and Hollister state that thus far only six cases of this form of interference with cavities have been reported, and only one, their own case, was successful in so far that it terminated in complete recovery. The clinical histories of these several cases are communicated in this paper, the original case being one of suppuration around a large echinoccocus cyst in the lung of twelve years' standing. An incision was made in the third intercostal space anteriorly through which the large cyst was subsequently removed. A counter opening being made between the fifth and sixth ribs, a drainage tube was introduced, and daily injections of carbolic acid practised. The authors conclude that "the operation is justifiable in any case where the presence of a gangrenous or ichorous cavity having been ascertained, it is found that, notwithstanding an outlet through the bronchi for a portion of the contents of the cavity, it steadily fills up again, the partial evacuation does not relieve the patient, who gradually loses strength and progresses toward a condition of collapse, a steady or intermittent rise in temperature continues; the infection of the healthy portions of the lung from the decomposed contents of the cavity has commenced, or is evidently about to take place; the breath and expectoration continue fetid; absence of appetite; increasing weakness, with or even without fever, etc. These indications will enable any medical man with some clinical experience to determine, in the majority of such cases, when the disease has reached a point from which spontaneous recovery is impossible." At the same time it is observed that any cavity covered by the scapula, or situated within the supra-clavicular and infra-clavicular regions may at present be regarded as inaccessible. The immediate indications and details of the operation are fully discussed in this paper, as well as the methods and after-management, of an interesting class of cases otherwise not amenable of treatment.—*Am. Jour. Med. Sci.*, October, 1881.

